# Cannabidiol (CBD) Treatment for Severe Problem Behaviors in Autistic Boys: A Randomized Clinical Trial

**DOI:** 10.1007/s10803-025-06884-y

**Published:** 2025-05-24

**Authors:** Doris Trauner, Anya Umlauf, David J. Grelotti, Robert Fitzgerald, Andrew Hannawi, Thomas D. Marcotte, Caitlin Knight, Lauren Smith, Gisselle Paez, Jennifer Crowhurst, Alyson Brown, Raymond T. Suhandynata, Kyle Lund, Marlen Menlyadiev, Igor Grant

**Affiliations:** 1 Department of Neurosciences, University of California San Diego School of Medicine, 9500 Gilman Drive, La Jolla, CA 92093-0935 USA; 2 Department of Psychiatry, University of California San Diego School of Medicine, San Diego, CA USA; 3 Department of Pathology, University of California San Diego School of Medicine, San Diego, CA USA; 4 Center for Medical Cannabis Research, University of California San Diego, San Diego, CA USA; 5 Rady Children’s Hospital San Diego, San Diego, CA USA

**Keywords:** Cannabidiol, Autism, Self-injurious behaviors, Aggressive behaviors

## Abstract

Open-label and observational studies suggest cannabidiol (CBD) reduces problematic behaviors in autistic children. No controlled clinical trials have addressed safety, tolerability, and efficacy. We conducted a double-blind, placebo-controlled crossover study of plant-derived CBD (Epidiolex®) to determine safety, tolerability, and behavior effects in autistic boys. Autistic boys with severe behavior problems age 7–14 years were randomized to eight weeks of CBD up to 20 mg/kg/day and eight weeks of placebo separated by a four-week washout. Behavioral assessments were completed before and after each treatment phase. Plasma concentrations of CBD were quantified. Primary outcomes were changes in total score of the Repetitive Behavior Scale-Revised (RBS-R), Child Behavior Checklist (CBCL), and Autism Diagnostic Observation Schedule-2 (ADOS-2). Both groups improved on the RBS-R and CBCL, with no significant difference between groups. ADOS-2 scores improved in placebo group only, but this improvement disappeared when other medications taken by the children were included in the analyses. Blinded clinical impressions showed almost 2/3 of the participants had behavioral improvements with CBD; 1/3 showed either no change or improvement on placebo. A strong placebo effect was observed. CBD had an acceptable safety profile. We did not find CBD to be clearly effective at reducing the broad range of behaviors characterized by the primary outcome measures. There was clinically evident improvement with CBD in 2/3 of participants. A prominent placebo effect demonstrates the importance of placebo control in treatment studies. Medications taken for behavior may reduce blood levels of CBD and may affect outcome measures.

*Trial Registration* https://clinicaltrials.govNCT04517799 start date 6/1/2020.

## Introduction

Cannabis and other cannabinoids have long been reported to convey a number of health benefits, including improving sleep, reducing anxiety, ameliorating chronic pain, treating headache, and providing relief in other medical and psychiatric conditions (Arkell et al., 2023; Bilbao & Spanagel, 2022). Unfortunately, there has been a paucity of controlled research to support the efficacy in all but a small number of conditions (Crippa et al., 2011, Dahlgren et al., 2022 {anxiety}; Devinsky et al. 2018a, b; Hausman-Kedem et al., 2018; Szaflarski et al., 2018 {epilepsy}; Leweke et al., 2012 {schizophrenia}; Masataka, 2019 {social anxiety disorder}). There is some suggestion that CBD alone, or compounds containing high amounts of CBD might also have anti-anxiety (Crippa et al., 2011; Dahlgren et al., 2022; Masataka, 2019), anti-psychotic (Leweke et al., 2012), and anti-inflammatory (Masoumi et al., 2023) actions. There has been increasing interest in recent years in the use of cannabinoids, especially compounds containing a high concentration of cannabidiol (CBD), to reduce problematic behaviors in individuals with autism spectrum disorder (ASD), including irritability and aggressive behavior (Aran et al., 2019, 2021; Barchel et al., 2019; Bar-Lev Schleider et al., 2019; Hacohen et al., 2022; Silva et al., 2022). None of these reports used purified CBD however, and all contained varying amounts of tetra-hydrocannabinol (THC). Notably, THC was present in from 10:1 to 20:1 ratios in every study. It is thus not possible to differentiate the effects of THC, CBD or the combination of both in improving problematic behaviors in the above studies. There have been no published studies to date that evaluate effectiveness of purified cannabidiol (CBD) in autism spectrum disorder.

Cannabidiol (CBD) accounts for about 40% of the cannabinoids found in the *Cannabis sativa* plant. Unlike the other major psychoactive cannabinoid found in cannabis, delta-9-tetrahydrocannabinol (THC), CBD is not intoxicating. Purified CBD (Epidiolex®) is now approved by the US Food and Drug Administration (FDA) to reduce seizure frequency in children with severe refractory epilepsies of childhood onset (Lennox-Gastaut syndrome, Dravet syndrome, or tuberous sclerosis complex in patients 1 year of age and older). CBD is thought to exert its effects by a number of mechanisms, including effects on multiple neurotransmitter receptors (Russo et al., 2005; Soares et al., 2010), possible effects on mitochondrial function (Masoumi et al., 2023; Unlu et al., 2023), activation of transient receptor potential channels (TRPV) that may reduce pain and modulate other sensory responses (Etemad et al., 2022); anti-oxidant (Jitca et al., 2023; Unlu et al., 2023) and anti-inflammatory properties (Jitca et al., 2023; Masoumi et al., 2023; Silva-Cardoso et al., 2023; Unlu et al., 2023).

Autism Spectrum Disorder (ASD) is a neurodevelopmental disorder affecting social development and communication as well as a broad spectrum of other symptoms. Children with ASD and severe behavioral problems, such as aggression and self-injurious behaviors (SIBs), present some of the most challenging problems. Their behaviors may be dangerous to themselves or those around them and may interfere with their ability to participate in school and play activities. Medications that are approved for use aim to reduce aggressive and irritable behaviors but can sometimes cause serious side effects (e.g., weight gain, diabetes, hypertension, tardive dyskinesia), and parents are at times reluctant to use them because of concerns about side effects. In recent years there have been several reports of “CBD” treatment reducing irritability and improving behavior in children with ASD (Aran et al., 2018; Barchel et al., 2019; Bar-Lev Scheider et al., 2019). Most have been observational studies, with a mix of CBD-containing products from dispensaries, all with THC as a prominent component. The studies have mostly been open label, lacking in placebo, and utilizing parent report to determine efficacy. While these reports have provided some suggestion that CBD-containing formulations may be useful in treating problem behaviors in children with ASD, they have not demonstrated a clear benefit of CBD because the products used had significant THC concentrations as well as other components of the cannabis plant that make it difficult to determine the relative benefits of CBD, and because of the lack of placebo-control and blinded data. Two controlled studies have shown mixed results. Silva et al. (2022) used a very low dose (unspecified concentration—6 drops/day of a CBD compound with 9:1 CBD:THC ratio) and found improvements in multiple areas including social interactions, agitation, and eating habits, using a semi-structured interview and the Autism Treatment Evaluation Checklist. However, Aran et al. (2021) using 2 formulations of CBD:THC each with 20:1 ratio, found mixed results, with no significant benefit on the primary outcome measures, but significant improvement in the CBD-treatment group on the Clinical Global Impression of Improvement (co-primary outcome measure) and the Social Responsiveness Scale (secondary outcome measure). Most studies to date involving participants with autism have included participants with few if any impairments in communication who are able to cooperate with study procedures. However, many of the more functionally disabling behaviors are observed among people with moderate to severe autism, for whom there is a paucity of clinical research information.

Because of the paucity of effective and safe medications for the problem behaviors often associated with ASD and given the earlier open-label reports of improvement in such behaviors with CBD-containing compounds, this study was designed to explore the question “Does a purified CBD formulation reduce problem behaviors in children with autism who have a high level of behavioral dysfunction?” using a double-blind, placebo-controlled, crossover design. We aimed to focus on children with moderate to severe autism who often require a high level of support and/or assistance for most or all activities of daily living.

The primary goals of the study were (1) to evaluate the safety and tolerability of orally administered purified CBD in boys with moderate to severe autism, and (2) to assess whether this CBD formulation alleviates adverse behavioral symptoms of autism (self-injurious behaviors, aggressive behaviors, extreme hyperactivity, and persistent stereotypies).

## Methods

### Study Design

**Fig. 1:**
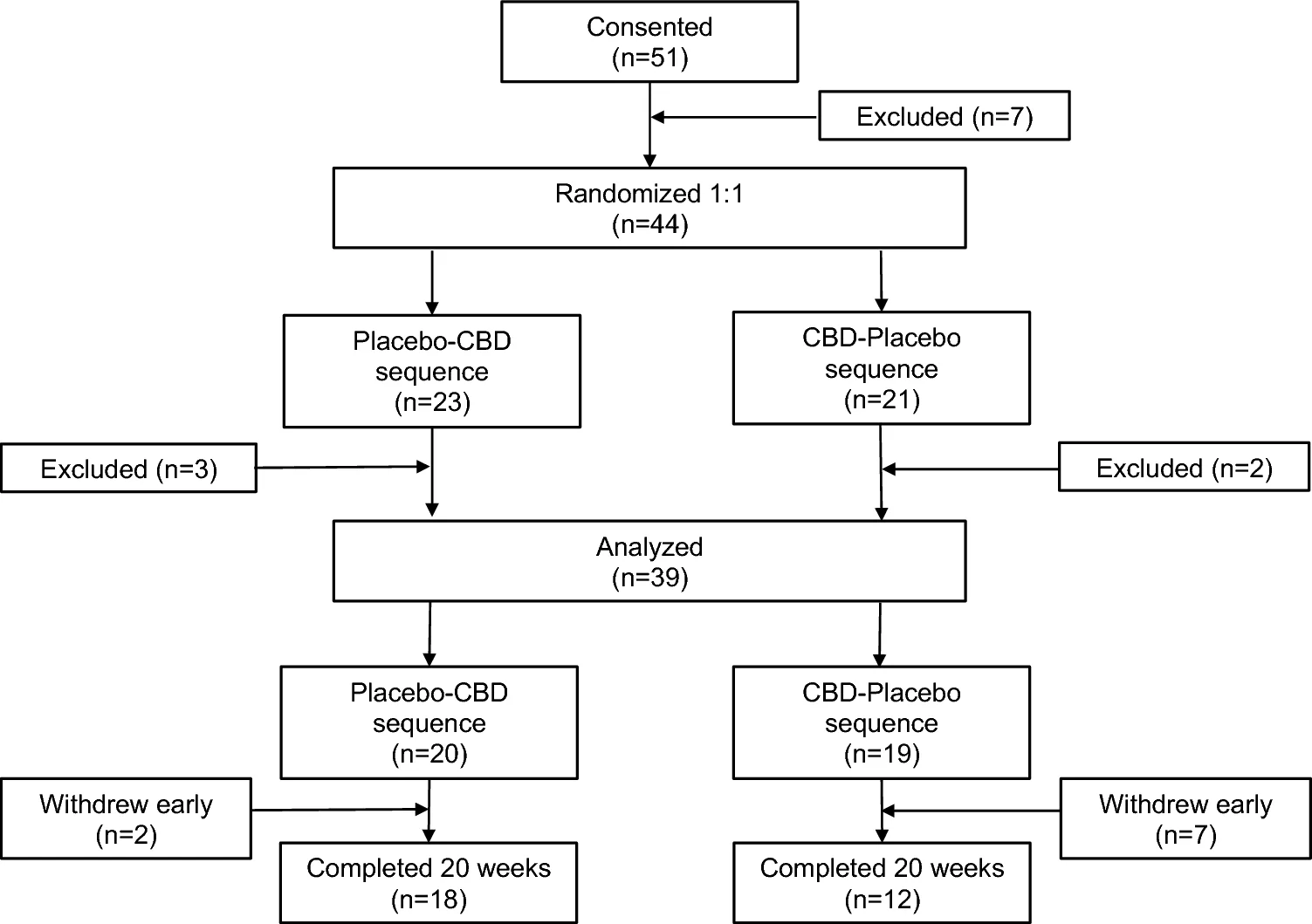
Study design flow chart. Baseline and repeat tests include ADOS-2, cognitive testing, blood tests, and parent questionnaires

The design was a double-blind, placebo-controlled, crossover study of 8 weeks of plant-derived purified CBD and 8 weeks of placebo. Boys with moderate to severe autism aged 7–14 years were randomized to placebo then CBD or CBD then placebo, using permuted block randomization method with block sizes of 2 and 4. Dosing of CBD was as follows: Week 1: 5 mg/kg/day, divided into 2 doses; Week 2: 10 mg/kg/day, divided into 2 doses; Weeks 3–8: 20 mg/kg/day, divided into 2 doses. An identical volume of placebo was used during the placebo phase in order to eliminate any bias about active drug vs. placebo in either phase of the trial. Participants received psychometric testing and parents responded to a set of questionnaires (see below) prior to receiving study drug. At the end of 8 weeks of treatment, each child was re-tested with the same tests as at baseline, and parents were asked to complete questionnaires. Treatment was then stopped for a “washout” period of 4 weeks, after which each child was re-tested and questionnaires completed again. Each child was then crossed over to either placebo or active drug, and the dose escalated again over 2 weeks, with a total treatment period of 8 weeks, after which time each child was re-tested and parental questionnaires completed (See Fig. 1).

The study drug was then discontinued, and the study completed after a final telephone follow-up safety check 2 weeks after the treatment phase ended.

### Subject Selection

Target enrollment for the study was 30 participants who completed all 20 weeks of the trial. Inclusion criteria were a confirmed diagnosis of autism on study entry using the Autism Diagnostic Observation Schedule-2 (ADOS-2—Gotham et al., 2007, 2009), free of other neurological conditions including epilepsy, and in general good health, with parent-report of moderate to severe persistent aggressive, self-injurious, and/or stereotyped and repetitive behaviors. We chose to limit this study to males. First, it has become increasingly apparent that girls with autism often demonstrate different behaviors than boys with this condition (Beggiato et al., 2017; Hartley et al., 2009; Antezana et al., 2019). Further, autism is recognized much more commonly in boys (~ 3:1 boys:girls-Loomes et al., 2017), possibly in part because of the differences in behavioral symptoms. Thus, in a sample size of 30, we would expect to recruit 7 girls meeting our criteria. Given the presumed sex differences in behavior and a relatively small sample size, we decided to limit this study to boys in order to reduce variability in the study sample.

Exclusion criteria were the following: the presence of epilepsy; a known genetic condition such as tuberous sclerosis; other significant health issues such as liver disease; presence of known congenital brain malformation; current use of CBD from another source, unless parents were willing to stop the treatment for at least 4 weeks prior to entering the study; known allergy to any of the components of the study drug; planned travel out of the area for a significant time during the study; or recent participation in another investigational drug trial. Children who were on medications known to interact with CBD such as clobazam or valproic acid were also excluded because of potential drug-drug interactions. Children were not excluded if they were taking a stable dose of a medication (e.g., risperidone, aripiprazole, guanfacine) for behavioral problems if the dose had not been changed for at least 3 months prior to entry into the study.

### Study Medication

Cannabidiol was administered in the form of Epidiolex®, an oral, plant-derived, purified CBD solution developed by GW Pharmaceuticals and FDA-approved for the treatment of seizures associated with Lennox-Gastaut syndrome, Dravet syndrome, or tuberous sclerosis complex in patients 1 year of age and older. A maximum dose of 20 mg/kg/day was chosen based on known safety, tolerability and efficacy of this medication in the treatment of intractable epilepsies. The CBD was donated by the pharmaceutical company along with placebo which was identical to the CBD in color, consistency, and taste. The company and its personnel had no role in the study and were not involved in the design or execution of the study, nor in data collection, data analysis or interpretation.

### Clinical Trial Methods:

Informed consent was obtained from a parent or legal guardian according to standards set by our Institutional Review Board. None of the children in the study were able to provide an assent because of significant cognitive limitations. Only after the consent was obtained were any tests administered; thus, some children who on pre-screening were thought to be eligible, had to be removed from the study after the initial testing showed they were ineligible.

A research pharmacist independent from the study used a predetermined randomization protocol to assign the participants to treatments, dispensed the study drug/placebo, and oversaw the crossover from the first to the second phase of the trial. All clinicians, study coordinators, psychometrists, and other personnel who interacted with the participants were blinded to treatment status. After the last participant completed his 20 weeks and all data were entered into the database, the blind was broken, and data were analyzed according to treatment.

Clinic visits: participants were examined in person in the Research Clinic at our institution at least every other week during the treatment phases. Vital signs were taken, adverse events recorded, blood draws performed for complete blood count, comprehensive metabolic panel, and cannabinoid levels, at each visit. Electrocardiograms were performed and a complete physical examination was completed. Clinicians noted whether there were any clinical changes such as improved or worsening behaviors.

To ensure that CBD was bioavailable when administered as an oral solution in this patient population and to demonstrate that participants were not receiving CBD or other cannabinoids outside of the study protocol, plasma specimens were collected and analyzed using liquid chromatography combined with tandem mass spectrometry (LC/MS/MS). These analyses were performed using similar techniques as were described previously (Hubbard et al., 2020). LC/MS/MS is considered the “gold standard” for making these kinds of measurements because it is both sensitive and specific. Blood was drawn between 30 min and 4 h after morning administration of the study drug. Parents were instructed to administer the study drug with a meal, and preferably with food high in fat, in order to increase absorption of the study compound.

### Primary Outcome Measures

Repetitive Behavior Scale-Revised (RBS-R) (Bodfish et al., 2000): This caregiver-completed scale examines the frequency and severity of repetitive and restricted behaviors. The RBS-R has been validated and shown to have high internal consistency and interrater reliability in outpatient settings. There are 5 subscales, including Stereotypic Behavior, Self-Injurious Behavior, Compulsive Behavior, Ritualistic/Sameness Behavior, and Restricted Interests. RBS-R Total Score was used as a primary outcome measure, with subscale scores analyzed as secondary outcome measures.

Child Behavior Checklist (CBCL) (Achenbach, 1991; Achenbach & Rescorla, 2001): the CBCL is a widely used parent/caregiver questionnaire that assesses problem behaviors in children. The school-aged scale used in this study consists of 118 questions. The CBCL consists of 8 syndrome scales, including Aggressive Behavior, Anxiety/Depressed, Attention Problems, Rule-breaking, Somatic Complaints, Social Problems, Thought Problems, and Withdrawn/Depressed. Total score was used as a primary outcome measure, with subscale scores as secondary measures. Analysis by subscales provides more specific information on the areas of interest in this study.

Autism Diagnostic Observation Schedule 2 (ADOS-2) (Gotham et al., 2007, 2009): This is a widely used tool for the diagnosis of autism spectrum disorders across individuals with a broad range of ages, developmental levels, and language abilities. It is a semi-structured assessment and consists of behavioral observation and coding of relevant behaviors. The ADOS-2 evaluates communication, social interaction, and imaginative play. There are 4 modules, based on level of verbal ability and age. Scores above 7 are consistent with autism; between 4 and 7, autism spectrum; and below 4, non-autism spectrum. The appropriate module of the ADOS-2 based on level of verbal ability was administered to each participant. The ADOS-2 was administered by trained testers in person, although masks were worn because of Covid-19 pandemic requirements.

### Statistical Analyses

Primary intent-to-treat analyses were conducted using mixed effects models with subject-specific random effects and fixed effects for treatment (placebo or CBD), study visit (2 or 3 per treatment), their interaction, and study period (first or last 8 weeks). Mixed effects modeling method was chosen for several reasons, including the need to account for the intra-class correlation of the longitudinal data from cross-over design, its advantages over other longitudinal methods when sample size is small, and its ability to utilize data from subjects with incomplete records. Data from every eligible child enrolled in the study were included in the analyses, including those who dropped out early. The primary analyses were not restricted to those who had completed the entire 20 weeks, because of such strategy’s potential to yield biased estimates and reduced statistical power (Fitzmaurice et al., 2011; Friedman et al., 2015). A significant treatment-visit interaction would be considered as evidence of CBD effect. Cohen’s d and its 95% confidence interval (CI_95_), based on the model’s estimates, were used to estimate effect size (ES). Cohen’s d is reported for change in an outcome over the 8-week treatment period (negative d represents improvement), separately for placebo and CBD, as well as for the difference in these changes between the two treatments (negative d represents stronger effect in CBD). Due to concerns of the lingering CBD effect that may not have completely cleared during the wash-out period, a sensitivity analysis was performed using data from the first 12 weeks only, with all measurements taken prior to crossover occurrence. For these analyses, the fixed effects in the mixed effects models included treatment, study visit (3 time points: baseline and weeks 8 and 12), and their interaction. One participant tested positive for CBD at the end of their placebo period (week 20). Per protocol analyses were conducted using methods described above after removing this participant’s data collected under the circumstances that deviated from the protocol. These analyses did not yield results different from the intent-to-treat analyses and, thus, the per protocol analyses results are not presented in this manuscript.

Prior to conducting the study, power analyses were carried out with simulations (k = 5000) using mixed effects model to estimate effect size for the treatment-by-time interaction term. The simulations estimated Cohen’s d = 0.40 or larger, assuming intra-class correlation ≥ 0.1, 80% power, α = 0.05, and total n = 27 (after hypothesized 10% dropout).

Group comparisons were performed using two-sample t test or Fisher’s exact test as appropriate. All analyses were performed using R version 4.2.1. P values < 0.05 were considered significant. Adjustments were not made for multiple testing.

**Fig. 2:**
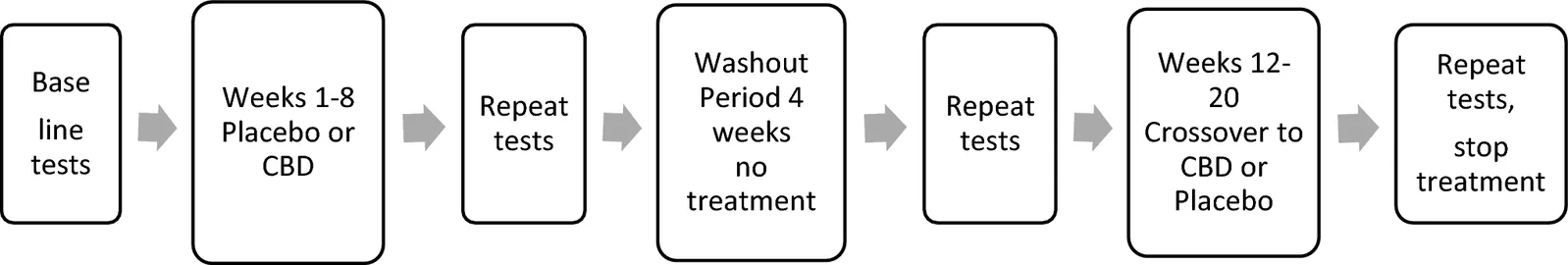
Participant flow chart

Although not a primary outcome measure, every participant was examined weekly or biweekly by a board-certified pediatric neurologist who recorded their clinical impressions of the child based on their direct examination and the information provided by parents regarding behavior for the previous week. These clinical impressions were reviewed once the blind was lifted (Fig. 2). Ratings of no change, minimal/mild, moderate, or marked clinical improvement or worsening were assigned based on the clinician notes.

## Results

### Demographics

Fifty-one boys were consented and thought to be eligible for the study (Fig. 2). After initial baseline assessments, 7 children were excluded from further study. Of those, 5 were determined to be ineligible based on their scores on the ADOS-2. One child was found to be allergic to sesame seeds, and another child had severe hyperglycemia and was excluded due to the possibility of type 1 diabetes mellitus. Forty-four participants were randomized to begin with either placebo or CBD, but 5 were excluded prior to receiving the first dose of study drug because blood drawing was unable to be completed due to inability to cooperate. Thirty-nine children were included in the final analyses. Nine of those withdrew prior to completing all 20 weeks of study (see Table 1). Seven started with CBD first and 2 with placebo first. Of the 7 who started with CBD, 4 withdrew because the child’s behavior worsened after the study drug was stopped at the end of 8 weeks. Thirty children completed all 20 weeks of the study. Because of the intent-to-treat data analysis design, all children who had received study drug at least once were included in the analyses, so that the final number used for data analysis was 39.

**Table 1:** Reasons given by caregiver for early withdrawal from study (n = 10)

Participant #	Treatment arm	Withdrawal week	Reasons (per caregiver)
1	Placebo first 8 weeks	3	Reduced appetite, behavior change
2	Placebo first 8 weeks	8	First time seizure
3	Placebo first 8 weeks	15	Increase in self-injurious behaviors
4	CBD first 8 weeks	4	Parents wanted to start artisanal CBD (were convinced he was on active drug and had improved)
5	CBD first 8 weeks	7	“Not a good fit” (possibly more aggressive, anxious)
6	CBD first 8 weeks	18	Family moved to another country
7	CBD first 8 weeks	10	Increased aggression during “washout” period
8	CBD first 8 weeks	12	Increase in self-injurious behaviors and aggression once first treatment ended
9	CBD first 8 weeks	10	Father reluctant to continue until other children had completed the study
10	CBD first 8 weeks	15	Increase in self-injurious behaviors in placebo phase

### Safety and Tolerability

All the participants were able to take the study drug orally without difficulty. Parents did not report any major difficulty in administering the treatment. There was no significant change in weight between baseline and 8 weeks of treatment in either the placebo or CBD phase of the trial. No electrocardiographic (ECG) changes were identified. Side effects were generally mild and did not lead to stopping the study drug except for one early withdrawal from the study of a child whose symptoms appeared to worsen during CBD treatment.

A total of 118 adverse events (AEs) were reported during the trial. Fifty-nine were reported during the CBD phase, 49 while on placebo, and 10 during the 4-week “washout” phase (Tables 2, 3, and 4). There were no serious adverse events (SAEs) related to treatment. Two SAEs were reported unrelated to the study including a participant with a first-time seizure during the placebo phase of the trial before cross-over to CBD, and one child who sustained a severe dog bite and required an emergency room visit for treatment.

**Table 2:**
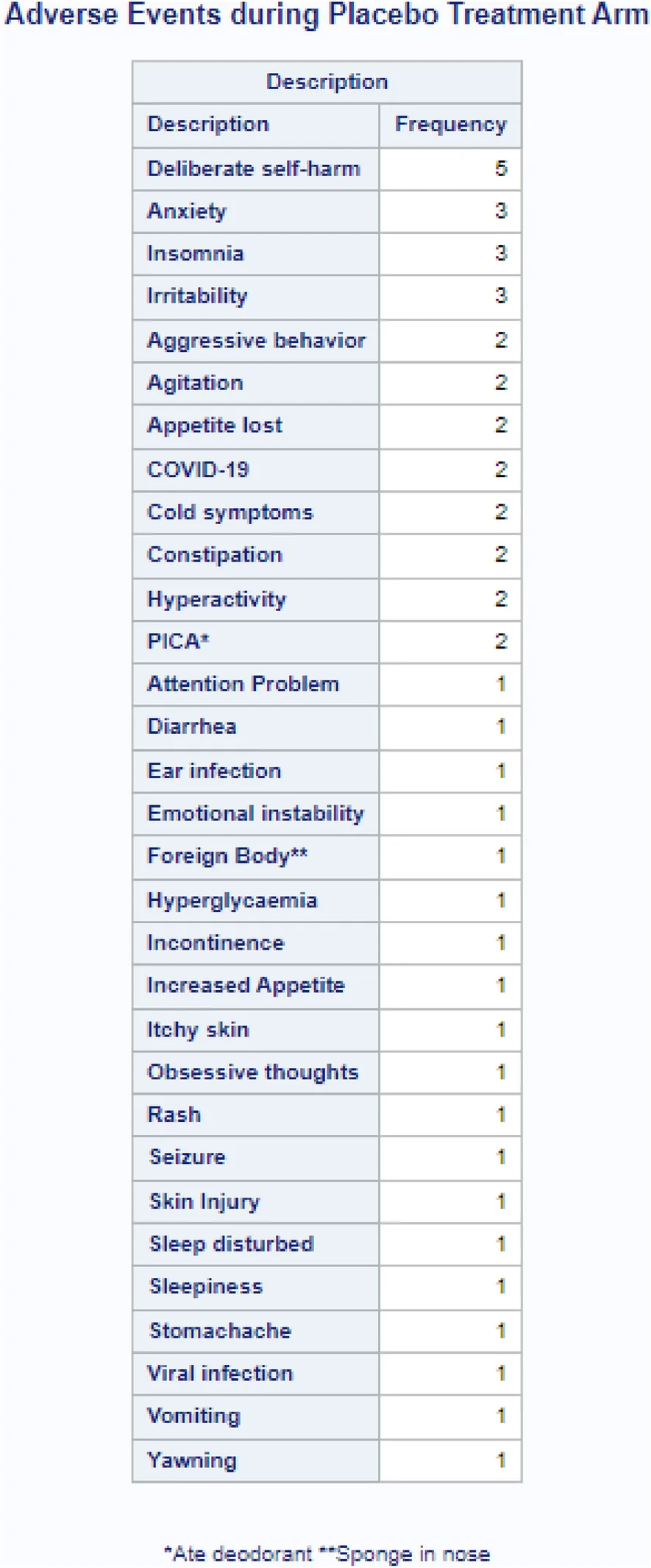
Adverse events reported by caregiver during placebo treatment arm

**Table 3:**
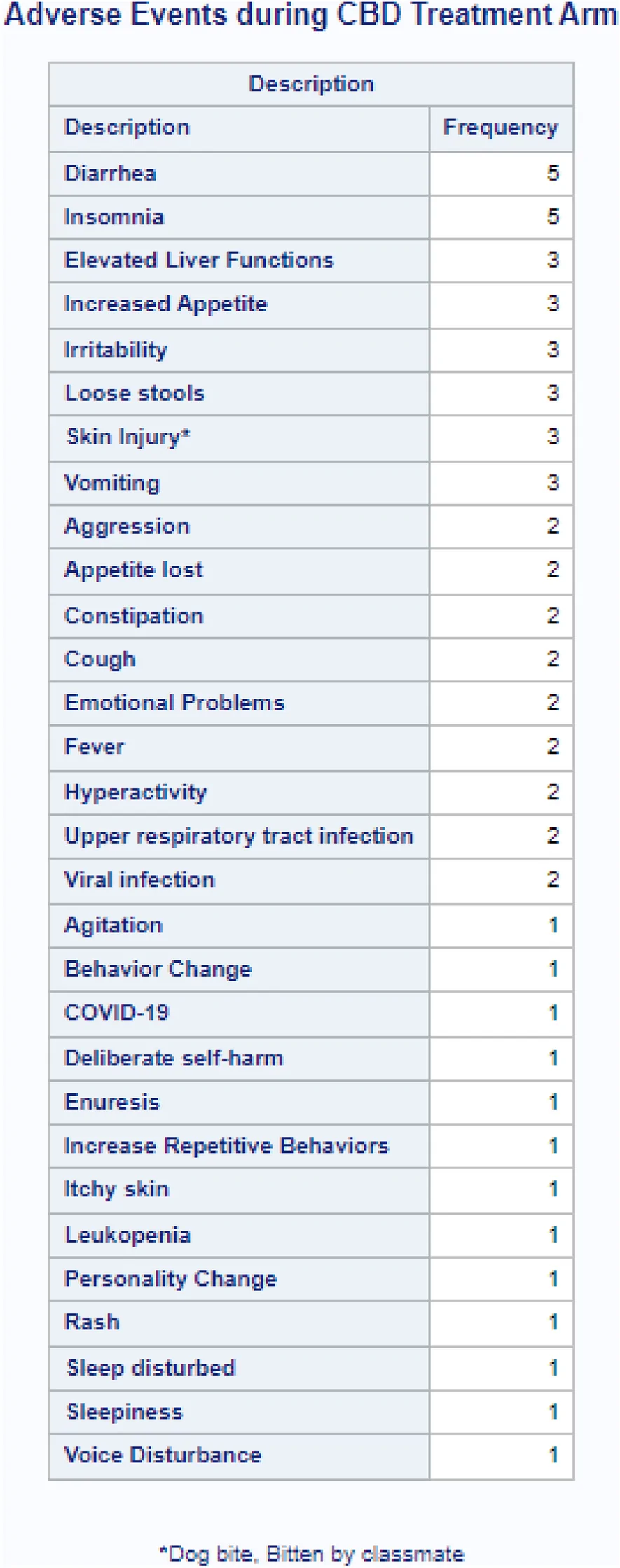
Adverse events reported by caregiver during CBD treatment arm

**Table 4:**
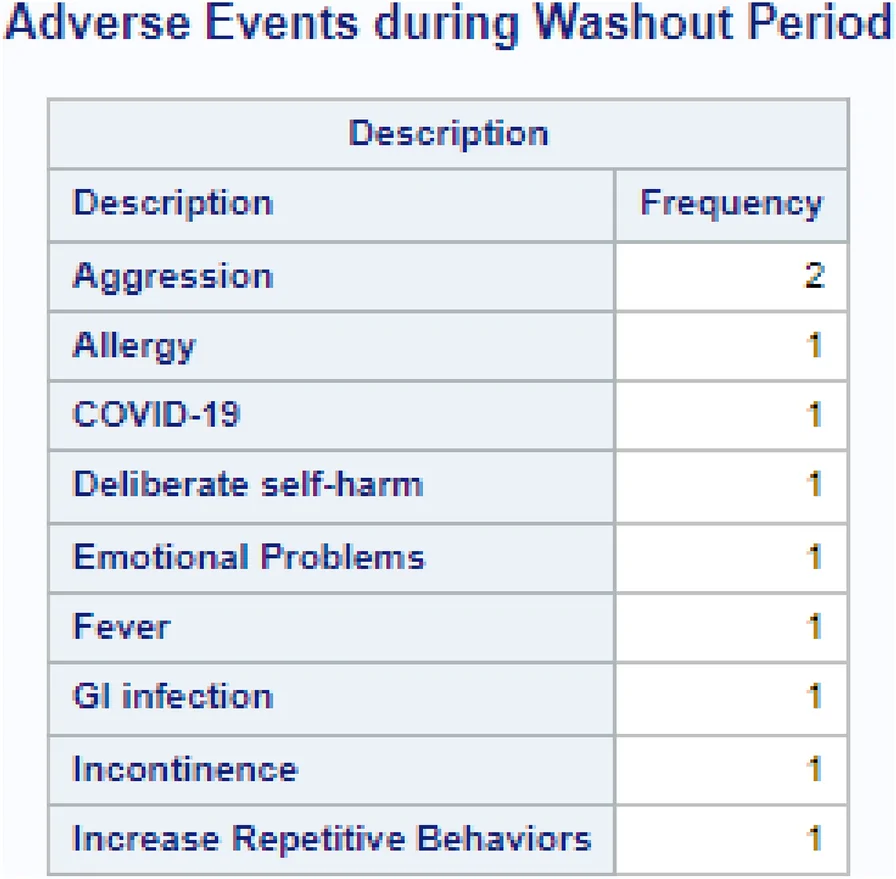
Adverse events reported by caregiver during washout period between the 2 treatment arms of the study (end of week 8 to end of week 12)

Figure 2 shows that at week 0 both the Placebo-CBD group and the CBD-Placebo group had no detectable levels of CBD or 7-OH CBD. As participants were up titrated with CBD to reach the target daily dose of 20/mg/kg day, plasma CBD concentrations increased approximately proportionally. For 31 participants who had data for the last visit of the CBD treatment arm, their individual average was calculated and then the group means were calculated from the individual values. For the CBD-Placebo group the mean (SD) CBD concentrations was 112 (82) ng/mL with a 95% confidence interval of 69–156 ng/mL. For the Placebo-CBD group the mean (SD) CBD concentration was 103 (46) ng/mL with a 95% confidence interval of 77–129 ng/mL. When both groups were combined the mean (SD) of CBD was 108 (67) with a 95% confidence interval of 83–132 ng/mL. Of samples collected across the 12-h dosing interval, an average CBD concentration of approximately 108 ng/mL was reached around 6 to 8 weeks after the first CBD dose. The concentrations observed are similar to those reported in pediatric patients receiving CBD oral solution for treatment-resistant epilepsy (Wheless et al., 2019).

**Fig. 3:**
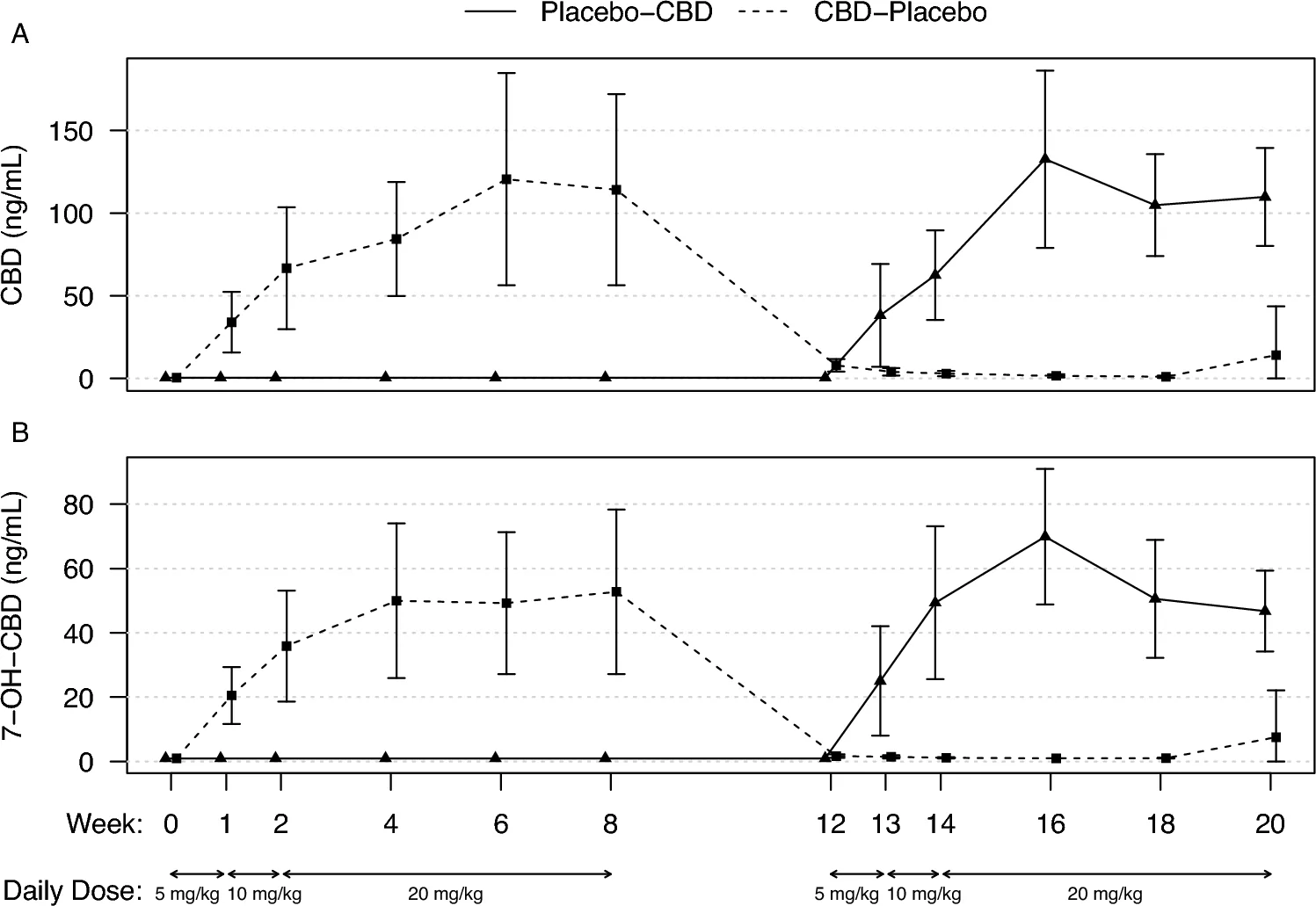
Concentrations of CBD and its active metabolite (7-OH-CBD) over the time course of the study. The wash-out period took place between study weeks 8 and 12

In a manner similar to the parent drug, the pharmacologically active metabolite 7-OH-CBD also increased during the dose titration (Fig. 3). For 28 participants who have data for the 3 last visits of CBD treatment, their individual average 7-OH concentrations were calculated and then the group means were calculated from the individual values. For the CBD-Placebo group the mean (SD) 7-OH-CBD concentration was 50 (37) ng/mL with a 95% confidence interval of 28–71 ng/mL. For the Placebo-CBD group the mean (SD) concentration of 7-OH-CBD was 52 (30) ng/mL with a 95% confidence interval of 35–70 ng/mL. When both groups were combined the mean (SD) concentration of 7-OH-CBD was 51 (33) with a 95% confidence interval of 38–64 ng/mL. This data demonstrates that the active metabolite was being formed and accumulated in this pediatric patient population.

No THC (or related compounds) were detected at any time point in any of the study participants.

One child who was receiving placebo in the second 8-week period was found to have measurable CBD (146 ng/mL) and 7-OH-CBD (73 ng/mL) concentrations at week 20 after having undetectable amounts for the previous 6 weeks. Since all study drug was turned back to us for destruction after each 8-week period, it is unclear how this occurred. We surmise that some small amount of study drug was retained by the family and administered at the end of the study period, but this is unsubstantiated. This event was recorded as protocol deviation. Per protocol analyses conducted after excluding this participant’s data did not yield results different from the intent-to-treat analyses and, thus, the per protocol analyses results are not presented in this manuscript.

### Primary Outcome Measures:

**Fig. 4:**
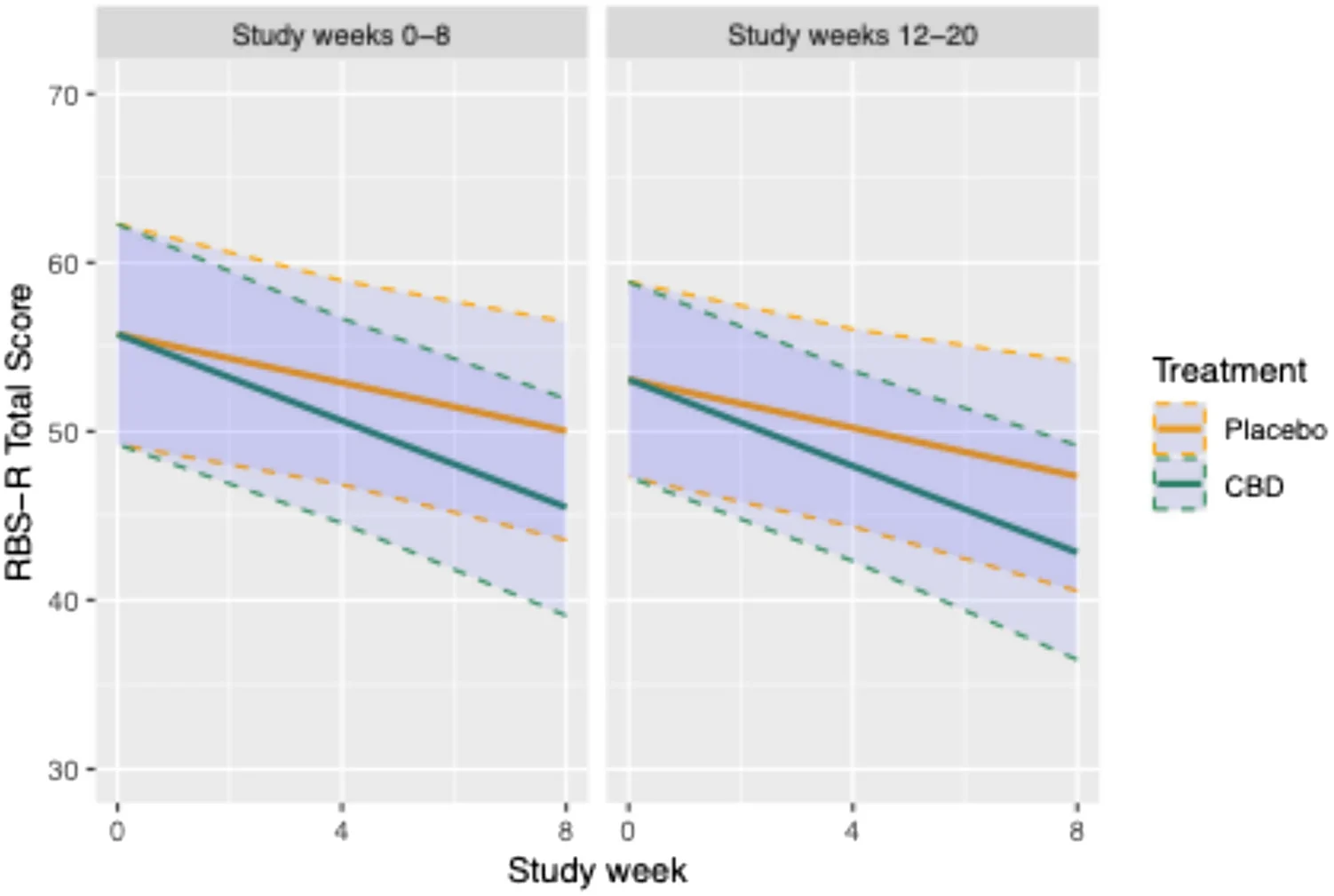
Predicted RBS-R Total Score estimated by the mixed-effects model; P-value for change in RBS-R Total Score .018 for Placebo, < .001 for CBD; effect size ~ 2 × greater for CBD than Placebo

On the RBS-R, between baseline and eight weeks of treatment, both placebo and CBD treatments showed significant improvements in Total Score (Fig. 4). When CBD and placebo groups were compared, the RBS-R Total Score showed a trend towards CBD over placebo treatment (d = -0.44; CI_95_ − 0.96, 0.07; p = 0.095). The CBCL Total Score showed similar improvements during the 8-week trial for both the placebo and CBD treatments, but there were no significant differences between the two treatments (d = 0.30; CI_95_ − 0.23, 0.83; p = 0.269).

ADOS-2 comparison scores varied to a small extent over the 2 treatment periods. During the placebo treatment arm, the ADOS score at baseline was (mean ± SD) 8.63 ± 1.31 and was 7.97 ± 1.66 at 8 weeks. For the CBD arm the ADOS baseline score was 8.30 ± 1.54 and 8.18 ± 1.45 at 8 weeks. These changes were not clinically significant and did not differ statistically between the two different treatment arms (p = 0.115). However, there was a significant reduction in the ADOS score for the placebo (d = -0.70; CI_95_ − 1.20, − 0.21; p = 0.007) but not for the CBD treatment arm.

Interaction between other medications used and outcome measures: Participants’ medication use, as reported at study’s baseline, was recorded and categorized based on type of medication (Table 5). A total of 27 (69.2%) participants were reported to use one or more of the following types of medication: alpha-2 adrenergic receptor agents, antihistamine/allergy aid, antipsychotics, benzodiazepines, constipation aid, mood stabilizers, SSRIs, sleep aid, stimulants, dietary supplements, others. The most frequently used medication types were sleep aids (n = 10), antipsychotics (n = 9), alpha-2 adrenergic receptor agents (n = 7), and dietary supplements (n = 6). There was no significant difference in the number of participants with medication use between treatment sequence groups (Placebo-CBD sequence: n = 12; CBD-Placebo sequence: n = 15; p = 0.301). There also was no significant difference (Placebo-CBD sequence: n = 8; CBD-Placebo sequence: n = 9; p = 0.751) between these groups on the number of participants using one or more of the 5 types of medication with most likely potential to affect behavior (alpha-2 adrenergic receptor agonists, antipsychotics, mood stabilizers, SSRI, and stimulants).

**Table 5:** Medication use by sequence, as reported at the beginning of the study

Type	Placebo-CBD	CBD-Placebo	Total
Sleep aid	6	4	10
Antipsychotic	4	5	9
Supplements (multivitamins, fish oil, prebiotics, other vitamins)	2	4	6
Alpha-2 adrenergic receptor agents	3	4	7
Mood stabilizer	1	2	3
Antihistamine/allergy	0	2	2
SSRI	2	1	3
Benzodiazepines	1	2	3
Stimulant	2	0	2
Constipation aid	1	2	3
Other	2	2	4
Total number of participants using antipsychotic, SSRI, Alpha-2 receptor agonist, stimulant, or mood stabilizer	8	9	17
Total number of participants using any medication	12	15	27

Baseline values of the three primary outcomes were compared by medication status. RBS-R total score did not differ (p = 0.69) between those using any type of medication (mean (SD) 56.4 (22.5)) and those not using them (59.5 (21.6)). It also did not differ based on use of the 5 likely behavior-affecting medication types (58.2 (26.5) vs 56.8 (18.4); p = 0.85).

CBCL total score at baseline also did not differ (p = 0.30) between those using any type of medication (68.1 (7.57)) and those not using them [65.2 (9.52)] or between those using 5 behavior-affecting types (69.8 (7.46)) and those not using them [65.2 (8.37); p = 0.08].

No statistical difference was found for baseline values of ADOS-2 comparison score based on any medication use [8.48 (1.16) vs 8.92 (1.38); p = 0.30] or based on use of the 5 behavior-affecting types [8.29 (1.21) vs 8.86 (1.21); p = 0.18].

The analyses for the primary outcomes were repeated with medication use as covariates. Specifically, we assessed use of any medication, use of any of 5 behavior-affecting types of medication, and use of specific types of medication for the top 4 most frequently used types. Three-way interactions of medication use, treatment, and time on the outcomes were tested. In addition, models for each treatment were repeated with medication use as covariates. These analyses did not yield additional findings in most cases. For ADOS-2, the results still did not show significant differences between CBD and Placebo effects. But we found a stronger CBD effect in the subset of participants who were not reported to use any of the 5 behavior-affecting types compared to the analysis on the whole sample. In this subset, the models show a decrease in ADOS-2 scores with CBD treatment (d = -0.95; CI_95_ − 1.71, − 0.18; p = 0.020) and with placebo, but without statistical significance (d = − 0.54; CI_95_ − 1.32, 0.24; p = 0.186).

**Fig. 5:**
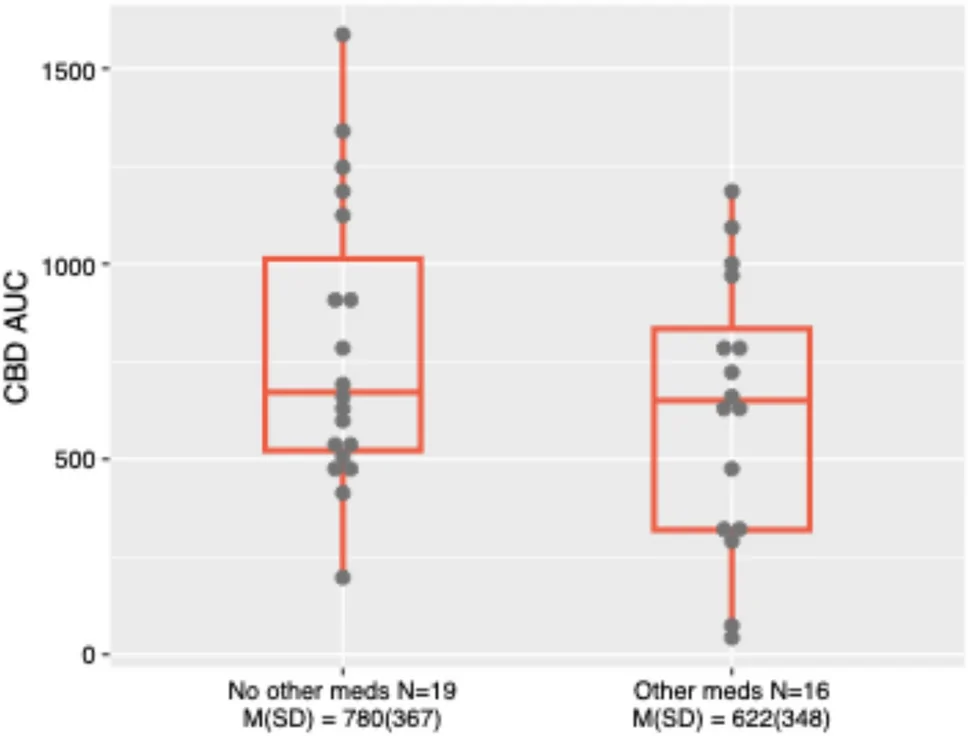
CBD concentrations (area under the curve—AUC) in children taking other medications at the onset of the study compared with those on no additional medications. CBD levels were lower in the children on other medications, although the differences were not statistically significant

When CBD blood levels (area under the curve for the entire 8-week period) were analyzed against medication use, a pattern of lower blood levels with any of the medications thought to affect behavior showed on average lower CBD levels for those using one or more of the 5 types of medication (antipsychotic, SSRI, alpha-2, stimulants, or mood stabilizer) (Fig. 5).

Clinical observations: based on blinded clinician impressions during clinic visits, 21 of the 31 children who completed all 20 weeks of the trial (68%) were correctly identified as to the phase in which they were taking active drug. Observations included less aggression (9), less hyperactive/calmer (8), improved communication/started to talk (6), fewer self-injurious behaviors (3) and lower anxiety (1). Of the 10 children who were mis-identified as being on CBD when they were on placebo, all but one had been on placebo the first 8 weeks. All but one showed some indications of improvement: decreased aggression (5), more communicative (3), calmer (2), and fewer self-injurious behaviors (1). The 3 children who had some improvement in communication during the placebo phase continued to improve further during the subsequent CBD phase.

## Discussion

This study was designed to examine whether a purified CBD compound had an acceptable safety profile and was well tolerated in boys with severe autism who required constant supervision and exhibited frequent and often severe negative behaviors, and whether CBD was effective in reducing problem behaviors such as aggressive, self-injurious, or repetitive and stereotyped behaviors, in those children compared to placebo. We found that this CBD formulation did have an acceptable safety profile and was well tolerated in this population. There were no significant effects of CBD over placebo on the primary outcome measures suggesting no global benefit of CBD in the treatment of boys with autism and severe behavioral problems. There was a prominent placebo effect found on all measures that may have dampened the ability to identify differences between groups. We did observe that improvements on the RBS-R were almost two times greater in the CBD than in the placebo group (Fig. 4), but the differences between groups did not reach significance. Since many of the children in this study had repetitive and self-injurious behaviors, we anticipated that there might be improvements in this score with CBD. The fact that both groups improved demonstrates the placebo effect, but the CBD group improved almost twice as much as the placebo group, suggesting that in a larger study group, differences would have been more obvious. Given that the number of participants was modest, the measures chosen were likely insufficiently sensitive to identify differences between groups. The use of the ADOS-2 as an outcome measure was chosen because of the severity of impairment in the study population, with the hope that it might provide a tool to identify global change with treatment. This was likely too broad an assessment measure and not suitable for monitoring changes in outcome.

Of note, however, were the clinician impressions of change in the group who completed the study. Approximately 2/3 (68%) of the participants who completed all 20 weeks were noted to have evidence of clinical improvement with CBD by blinded clinicians who saw the children on a weekly or biweekly basis throughout the study. Reductions in aggressive behaviors and hyperactivity were observed most frequently, with improvements in communication being seen in almost 30%. Since many of the participants were non-speaking at the onset of the study, this was a notable change, with several of the children beginning to use words for the first time.

A second pertinent finding was a review of the reasons parents reported for removing their children from the study prematurely. Of the 10 children who ended the study prior to 20 weeks, two stopped early for reasons unrelated to the study (first time seizure, family move). Four of the remaining 8 were removed for increasing problem behaviors noted after the CBD phase ended, suggesting that there had been an improvement in those behaviors while on CBD. Three had worsening behaviors while on placebo and 2 while on CBD. Taken together, the clinical impression and parent report data suggest that CBD may be beneficial for a subset of children with severe autism. It is unlikely that the observed worsening of behavior is related to withdrawal. Abrupt discontinuation of CBD has not been associated with withdrawal effects after a 4-week treatment in healthy volunteers (Taylor et al., 2020). However, it is not known if and how withdrawal effects might manifest in children with autism and severe behavioral problems.

Biomarkers or other variables able to identify subsets of children with autism who might benefit from CBD are needed to guide treatment planning and parent counseling.

Participants were allowed in the study if they were on stable doses of other medications for at least 3 months prior to entering the study. Interestingly, we found that children who were taking any of 5 categories of “behavior” medications (i.e., ones that are commonly used to treat problematic behaviors in children with autism) had lower overall CBD blood levels than those who were not on any medications. Also, instead of tending to favor placebo, when medication use was taken into account, we now found ADOS-2 improvement on CBD and no significant difference between CBD and placebo on ADOS testing.

Although the results were not significant, likely due to the low number of participants, this is an area that deserves further study with a larger group of participants, as it may provide one explanation for absence of response to CBD in some children and not others. Our numbers were too low to determine whether this might be the case.

This study also demonstrates the crucial importance of having a placebo group when trying to determine the efficacy of a novel treatment for a condition as complex as autism. We found a robust placebo effect, which would not have been appreciated without a placebo control group. Previous reports of improvement in behaviors of children with autism using cannabinoids have largely been observational, without controlling for dose or specific formulation of the product, with use of THC-containing formulations, and often without a control group. Our results call into question the value of such open label studies and stress the importance of families and other raters being blinded to treatment arm.

There has been increasing interest in placebo effects identified in clinical trials of autism in recent years (e.g., Aran et al., 2023; Curie et al., 2023). Several double-blind, placebo-controlled studies have failed to demonstrate superiority of study treatment over placebo, despite anecdotal, observational, and open-label studies finding improvements. This was notably the case with secretin, for which there were claims of major improvements in autism symptoms, but which failed to show superiority over placebo in blinded trials (Krishnaswami et al., 2011; Owley et al., 2001). Similar negative results have recently been found for oxytocin (Daniels et al., 2023; Guastella et al., 2023), citalopram (Simonoff et al., 2022), and bumetanide (Fuentes et al., 2023). Clinical trials in autism are often based on ratings from caregivers and clinicians and be vulnerable to “placebo effects by proxy”. Like secretin (Grelotti & Kaptchuk, 2011), placebo response in this study may have been particularly large because parental expectations were likely influenced by positive media reports about the many potential health benefits of CBD. The FDA approval of CBD for rare pediatric epilepsy syndromes that cause severe neurodevelopmental delay might also have raised parental expectations that CBD would help children with severe autism. Although parental expectation was not associated with placebo response in a trial of a mixture of CBD and THC for children with autism [Aran et al., 2023], societal views about administering THC to children are likely less positive than for CBD and may have tempered expectations of a benefit of combining CBD and THC. In order to mitigate placebo effects by proxy and other influences of expectation, investigators should include objective measures of treatment response as outcomes, in particular where there is already significant belief in the efficacy of the treatment based on anecdotal or media influences (Grelotti & Kaptchuk, 2011).

Possible favorable perceptions of the health effects of CBD may not override negative parental expectations about possible side effects. In our study, the number of adverse events during the placebo arm was similar to the number during the treatment arm. Parental worry over medication-related side effects, particularly in the context of a clinical trial, might influence perceptions of their child’s health and/or behavior. This again highlights the importance of a placebo group and more focused, objective measures of treatment effects.

One randomized controlled trial (Aran et al., 2021) of a formulation high in CBD but containing THC as well for treatment of autism found that there was insufficient evidence for efficacy because of mixed results, with questionnaire data showing no difference between groups, but Clinical Global Impression of Improvement (CGI-I) results showing more improvement in the CBD-treated group. Interestingly, although the 2 questionnaires used in their study were quite different from the ones used in the present study, our results were similar, confirming the importance of placebo control and blinding of all participants, but also the challenge of choosing the most appropriate tools with which to adequately assess changes in behavior in this very diverse population of children.

There are a number of limitations to this study. As a phase-2 study taking place during the Covid-19 pandemic, there was a modest number of participants who completed the entire 20 weeks of the study, and given the prominent placebo effect, a larger n might have demonstrated a stronger difference between the groups. We included male participants only; thus, we cannot make any statements about possible effects of CBD in autistic girls with severe behavioral challenges. Further, we included only boys with severe behavior problems and significant functional limitations; it is possible that CBD may exert a different effect in individuals who have milder levels of dysfunction. Only one dose of CBD was used in the study; thus we may have missed a dose-dependent effect of CBD on the targeted behaviors. As mentioned above, the primary outcome measures chosen for the study were likely not specific or sensitive enough to identify the types of improvements that we found with CBD over placebo. Also, in such a small sample, it is possible that unblinding related to elevations in LFTs (especially as this caused a change in study dose) and common side effects of CBD, such as diarrhea, which were more common in the CBD group here, may have biased the results in favor of CBD. The exposure time to CBD was relatively short (8 weeks). It is possible that a longer period of exposure might be necessary to identify changes in such an impaired population.

Finally, it is important to note that this trial took place at the height of the Covid-19 pandemic (2020–2022). This resulted in school closures, major changes in routines, home-schooling, reduction in therapy services, and other changes that may have altered our participants’ behavioral responses and created more variability. Restrictions and precautions necessitated that all personnel wear facial masks and eye protection when interacting with the study participants. This requirement may have altered the results of the testing; however, the same protective equipment was used throughout the entire study. Thus any alterations in test results produced by wearing such equipment would be expected to be present throughout the trial.

Despite the limitations, this study provides valuable new information about a potential role of CBD in improving behavior in a subset of children with autism, as well as the strong role the placebo effect plays in this condition. Importantly, CBD at a dose of 20 mg/kg/day appears to have an acceptable safety profile and is well tolerated in boys with autism. A study utilizing the same placebo-controlled and blinded approach with a highly purified and stable dose of CBD but with a larger number of children, more targeted objective measures of treatment response, and controlling for other medications used, powered sufficiently to examine efficacy in boys and girls, may be able to more definitively answer the question of whether CBD reduces problem behaviors in children with autism.
